# Baby Business: a randomised controlled trial of a universal parenting program that aims to prevent early infant sleep and cry problems and associated parental depression

**DOI:** 10.1186/1471-2431-12-13

**Published:** 2012-02-06

**Authors:** Fallon Cook, Jordana Bayer, Ha ND Le, Fiona Mensah, Warren Cann, Harriet Hiscock

**Affiliations:** 1 Parenting Research Centre 5/232 Victoria Parade East Melbourne, Victoria 3002 Australia; 2 Centre for Community Child Health, Murdoch Childrens Research Institute The Royal Children's Hospital Melbourne 50 Flemington Road Parkville, Victoria 3052 Australia; 3 Deakin Health Economics Deakin University 221 Burwood Hwy Burwood, Victoria 3125 Australia; 4 Clinical Epidemiology and Biostatistics Unit Murdoch Childrens Research Institute The Royal Children's Hospital Melbourne 50 Flemington Road Parkville, Victoria 3052 Australia

## Abstract

**Background:**

Infant crying and sleep problems (e.g. frequent night waking, difficulties settling to sleep) each affect up to 30% of infants and often co-exist. They are costly to manage and associated with adverse outcomes including postnatal depression symptoms, early weaning from breast milk, and later child behaviour problems. Preventing such problems could improve these adverse outcomes and reduce costs to families and the health care system. Anticipatory guidance-i.e. providing parents with information about normal infant sleep and cry patterns, ways to encourage self-settling in infants, and ways to develop feeding and settling routines *before *the onset of problems-could prevent such problems. This paper outlines the protocol for our study which aims to test an anticipatory guidance approach.

**Methods/Design:**

750 families from four Local Government Areas in Melbourne, Australia have been randomised to receive the *Baby Business *program (intervention group) or usual care (control group) offered by health services. The *Baby Business *program provides parents with information about infant sleep and crying via a DVD and booklet (mailed soon after birth), telephone consultation (at infant age 6-8 weeks) and parent group session (at infant age 12 weeks). All English speaking parents of healthy newborn infants born at > 32 weeks gestation and referred by their maternal and child health nurse at their first post partum home visit (day 7-10 postpartum), are eligible. The primary outcome is parent report of infant night time sleep as a problem at four months of age and secondary outcomes include parent report of infant daytime sleep or crying as a problem, mean duration of infant sleep and crying/24 hours, parental depression symptoms, parent sleep quality and quantity and health service use. Data will be collected at two weeks (baseline), four months and six months of age. An economic evaluation using a cost-consequences approach will, from a societal perspective, compare costs and health outcomes between the intervention and control groups.

**Discussion:**

To our knowledge this is the first randomised controlled trial of a program which aims to prevent both infant sleeping and crying problems and associated postnatal depression symptoms. If effective, it could offer an important public health prevention approach to these common, distressing problems.

**Trial registration number:**

ISRCTN: ISRCTN63834603

## Background

In the first six months of life, between 15-35% of parents report a problem with their infant's sleep [1-3] including difficulties settling their infant to sleep at the start of the night and re-settling them overnight. Such problems disturb parental sleep leading to parental fatigue [4], reduced ability to care effectively for the infant, and parental depression symptoms [5]. Persistent infant sleep problems are associated with later child behaviour problems [6-8].

Similarly, around 14-28% of parents report infant crying as a problem in the first few months of life and sleep and crying problems often co-exist. Crying duration exceeding 3 hours/24 hours for at least 3 days for at least 3 weeks is typically known as 'colic' and affects between 9-12% of infants from community samples [9,10]. Parents of crying infants may experience frustration and anger towards their infant and parental perception of crying as a problem is the most common proximal risk factor for Shaken Baby Syndrome [11]. Problem crying is associated with early weaning from breast milk, frequent changes of formulae and parent depression symptoms. Parental *perception *that their infant's crying is a problem therefore merits attention, regardless of whether the actual duration of crying meets criteria for 'colic'.

### Sleep problems in infants

Approximately two-thirds of infants learn to 'sleep through the night' i.e. experience unbroken sleep between the hours of midnight and 5 am, by 12 weeks of age [12,13]. However, up to a third do not [14] and may also have problems initiating sleep [15]. Such problems have been consistently linked to parental mood disorder [16,17] and when infant sleep problems are treated parental depressive symptoms decrease [18,19]. Preventing infant sleep problems may therefore be an acceptable way to reduce rates of parental depression, particularly given that breast feeding mothers are often reluctant to accept pharmacologic treatment for depression [20].

Infant sleep problems tend to arise when parents actively help their infant to fall asleep [21,22]. Infants, like adults, tend to wake briefly several times during the night, but parents are only aware of this if the infant cries out (signals) and wakes the parents. Parents who rock, feed, or remain with their infant while the infant falls asleep, are more likely to report frequent night awakenings and settling problems [21], as their infants tend to cry out and demand the same attention each time they wake during the night. Conversely, parents who allow their infant to settle to sleep independently with little caregiver interaction, report fewer immediate and long term sleep problems [23,24]. Their infants tend to be more successful at falling back to sleep without the caregiver's help throughout the night [15].

Maternal cognitions about infant sleep are also strongly related to infant sleep problems [25]. Mothers who have problems resisting their infant's demands, who feel anger and helplessness when faced with their infant's demands, or who feel doubt regarding the adequacy of their parenting, are significantly more likely to report infant sleep disturbance [25]. In order to cope with these feelings, parents may become 'over intrusive' at bedtime. This may result in the infant learning caregiver dependent sleep associations that in turn lead to more frequent night signaling [21].

### Crying in infants

Around 14-28% of parents report infant crying as a problem [9,26,10]. Infant crying duration peaks at around 6-8 weeks of age at around 2.5 hours per 24 hours, then gradually declines [27,28]. 'Colic' refers to crying in excess of 3 hours per day for at least 3 days per week, over a three week period that resists soothing. The cause(s) of colic are unknown. Although long attributed to gastrointestinal upset (e.g. flatulence and gastro-oesophageal reflux) [29,30] and organic disturbances such as food intolerance or allergy [31], medical causes are thought to affect only one in ten infants [32]. Yet many parents change their own diet (if breast feeding) or the infant's formula, in the belief that a food intolerance underlies the crying problem [33,34]. Others seek over the counter medications for reflux or alternative therapists such as chiropractors, both of which have been shown in rigorous trials to have no impact on crying [35,36]. Other medications are either ineffective (e.g. simethicone) [37,38], associated with serious side effects (e.g. dicyclomine and apnoeas) or may predispose the infant to harm (e.g. acid suppressive medications and eosinophilic oesophagitis)[39].

Parenting style may also affect the amount of fussy crying (crying that can be soothed) as well as colicky crying (crying that is unsoothable). 'Proximal care' describes a parenting style that typically involves feeding an infant in excess of 14 times per day, holding them for greater than 80% of the day time, and cosleeping (i.e. parent sharing a sleeping surface with the infant). One study compared the impact of this approach on infant sleep and crying with a more structured approach (i.e. feeding infant every 3 to 4 hours, placing them in a cot for sleep and a delayed response to infant demands) and an approach somewhere between the two styles [40]. Comparison of approaches revealed that proximal care parents had infants with less fussy crying but the same amount of colicky crying as the more structured approach. Proximal care resulted in more frequent waking and crying at night at 12 weeks of age. The authors concluded that a proximal care approach throughout the first few weeks of life may be useful in reducing overall amounts of non-colicky crying, but changing to a structured approach after this time may result in less night waking at 12 weeks of age [40]. Any program which aims to prevent early infant sleep and cry problems could incorporate this approach.

### Interventions for existing infant sleep/cry problems

Behavioural strategies such as graduated extinction (where the parent returns to check on their crying child at increasing time intervals with brief parental reassurance) and positive bedtime routines [41,42] have been shown to be the most effective strategies for managing sleep problems in children aged 6 months or older. Despite the effectiveness of such interventions [43,44], some parents find techniques involving extinction unacceptable, as they dislike leaving their infant to cry [25,42]. Given that these parents may not follow through with strategies, it may instead be preferable to aim to *prevent *sleep problems. Prevention may have additional advantages including greater efficacy (as problem is less entrenched) and prevention of associated parental distress.

Few randomised controlled trials (RCTs) have evaluated interventions for colicky infant crying. Most have involved changes to diet or use of medication, with results mostly suggesting no effect over and above placebo and additionally, the methodological rigor of these trials has been questioned [45]. Increased carrying of the infant has been shown in one trial to prevent crying [46] but did not reduce established crying in another trial [47]. Keefe [48] has proposed a model to explain infant colic as a psychobiological disturbance in infant behaviour regulation due to increased sensitivity to the environment. Disruptions or inconsistencies in parenting or the surrounding environment overstimulate the infant resulting in crying that the infant does not yet have the maturity to regulate. With this in mind Keefe and colleagues [48] conducted a RCT of an intervention for colicky infants aged two to six weeks. In the intervention group (n = 64), nurses visited families four times over one month to provide support to parents, make modifications to infant care (with an emphasis on consistency of routines) and educate parents on reducing overstimulation in their infant. The control group (n = 57) received usual care. Compared with the control group, intervention infants had a significantly higher number of resolved crying problems (61.8% vs. 28.8%, p = 0.03) as well as shorter total crying time/day (1.29 vs. 2.94 hours, p = 0.02) at approximately 13 weeks of age (exact mean age not given in manuscript). This suggests that intensive parental support, modification of environment and provision of structured care can reduce crying.

### Can we *prevent *infant sleep and cry problems?

Only four RCTs have examined the impact of prevention programs on infant sleep problems. No RCTs have aimed to prevent both infant sleep *and *cry problems. In a small RCT of middle-class first time parent couples (intervention n = 29, control n = 31), Wolfson and colleagues [24] provided parents with two prenatal and two postnatal group sessions that taught parents about normal infant sleep/wake patterns and the importance of establishing an independent sleep routine so that the infant can self-settle. Infants of parents who attended these group sessions slept for longer amounts of time, and demonstrated better sleep patterns than infants of control group parents at one, two and three weeks of age.

Kerr, Jowett & Smith's [49] RCT aimed to prevent infant sleep problems by providing parents with information on settling methods and the importance of routine via a booklet and a home visit (intervention n = 86 and control n = 83). Information was provided at three months of age and follow up data were collected at nine months. Compared with control group infants, intervention group infants had significantly fewer settling difficulties (21% vs. 39%, p = 0.03), significantly fewer night awakenings (23% woke two or more times per night vs. 46%, p = 0.02) and significantly better cumulative sleep scores overall (22% met criteria for a severe sleep problem vs. 39%, p = 0.03).

In a three-armed RCT that aimed to prevent infant sleep problems in infants aged 8 to 14 days [23], families were allocated to receive either (1) a structured behavioural program (n = 205), (2) an education oriented group (n = 202), or (3) the usual care provided by UK health services (n = 203). Parents in the behavioural group were asked to allow their infant to settle to sleep independently, to only respond to the infant when genuinely crying (as opposed to fussing or fretting), to keep stimulation low during the night, and after three months of age, to gradually increase the time between night feeds. Parents received the information via a flyer which a researcher also discussed with the parents. Parents in the education oriented group received the same information in a ten page booklet and the information was suggestive rather than prescriptive. By 12 weeks of age, significantly more behavioural group infants were sleeping through the night (having uninterrupted sleep from 12 am-5 am) than the other two groups. Behavioural but not education group parents tended to access significantly fewer health services for their infant's sleep in the following six months. In a post hoc analysis [50], infants receiving in excess of 11 feeds per day at one week of age were more likely to wake during the night at 12 weeks of age. The data of infants who met this criterion, from both the behaviour group, and the control group, were then compared. By 12 weeks of age, 80% of these 'at risk' infants in the behaviour program, compared to 60% in the control group, were sleeping through the night. Thus a behavioural program may be particularly useful for preventing sleep problems in infants who feed frequently in the first week of life.

In another RCT that aimed to prevent infant sleep problems, parents recruited via birth notices in the local newspaper were randomly allocated to receive either a 45 minute consultation with a nurse accompanied by written information at infant age three weeks (intervention group, n = 137), or usual care (control group, n = 131) [51]. Intervention group parents were taught about the cyclical nature of sleep and the benefits of parent-independent sleep cues. Parents were recommended to leave the infant to settle for five minutes before responding if the infant was crying and to extend this response time by five minutes for each subsequent visit. All parents completed a 7 day infant behaviour diary at 12 weeks of age. Intervention infants were significantly more likely to have at least 15 hours of sleep per 24 hours than control infants (62% vs. 36%, respectively, *p *< 0.001). The sample was predominantly middle-upper class thereby limiting the generalisability of the findings.

Prior research has been limited by the use of population sampling that is self-selected [51,24], excludes unmarried parents [24] or fails to collect data on compliance with intervention strategies [49,51,24]. Despite strong links between infant sleep and crying problems and parental depression [16,17], parent well being has rarely been included as an outcome [49,24]. Data from fathers is often lacking despite the increasing role fathers play in infant care [52] and the protective role they can play in prevention of postnatal depression in mothers [53-55]. Therefore, involvement of fathers in trials that have a specific focus on infant and mother wellbeing is paramount.

In summary, an infant demand style of approach in the first few weeks of life may reduce overall crying, but changing to a more structured style of care after these first few weeks, may result in less night waking at 12 weeks of age [40]. A program that implements elements of both approaches and provides parents with information about normal sleep and cry patterns and ways to reduce stimulation and provide a predictable environment in the first few months of life, may be able to prevent both infant sleep and cry problems [14,48]. Reduction in these problems is likely to have positive flow on benefits for parent wellbeing [18,19], and could lead to reduced health service use for both parent and infant wellbeing [23]. This paper presents the study protocol of a randomised controlled trial of a universal parenting program designed to prevent both infant sleep *and *cry problems, and improve parental wellbeing. The recruitment phase of this trial is currently complete and intervention delivery and follow up assessments are ongoing.

## Methods/Design

### Study Design

Randomised controlled trial.

### Setting

Four Melbourne (state of Victoria, Australia) Local Government Areas (LGAs) of Brimbank, Wyndham, Moonee Valley and Yarra. We selected these LGAs based on a Victorian government request to carry out the study in the north-western region of Melbourne and on annual birth rates in excess of 1000 to maximise recruitment.

### Participants

#### Inclusion criteria

All parents of newborn infants seen by their Maternal and Child Health Nurse (MCHN) at their first home visit (day 7-10 postpartum) in the four LGA's. The MCH nursing service is a universal, free service offered to all Victorian families with scheduled visits (covering 93% of all births) post partum, then at 2 weeks of age, and 1, 2, 4, 8, 12, 18, 24 and 42 months.

#### Exclusion criteria

Parents with insufficient English to complete the questionnaires and take part in the intervention have been excluded. Infants born before 32 weeks gestation or with a serious health concern have been excluded as program material may not be suitable for very premature or ill infants.

### Sample Size/Power calculation

In a Victorian survey of 724 mothers, [4] 34% reported sleep problems at mean infant age of 4.6 months (range 3-6 months). In a community survey of Queensland parents [56] 27% reported a problem in their 4-6 month old infants (n = 740). Drawing upon this data, a relative reduction in the prevalence of parent report of an infant sleep problem (sleep problem yes/no) by 30% at infant age four months (i.e. from 30% to 20%) is likely to be clinically significant and to have flow on effects for parent mental health. Assuming this, we aimed to detect a reduction in the proportion of infant sleep problems from 30% to 20% at 4-months with 80% power at the 5% significance level. In a trial that randomises individuals, we required 313 subjects in each arm or a total of 780 families, allowing for a 20% loss to follow up. Due to time and budget restraints, we have been able to recruit 750 families only, giving us 78% power to detect a reduction in the proportion of infant sleep problems from 30% to 20% at 4-months, at the 5% level of significance.

### Randomisation

Using a computer generated random number sequence, an independent researcher allocated each consenting family to the intervention or control group. The research team and families remained blind to group allocation at the time of recruitment and consent; however, following this, knowledge of group allocation is unavoidable given the intervention type.

### Reducing bias

Despite being at risk of response and subjective bias, parental *perceptions *of either a sleep or cry problem (yes/no) is our most important outcome measure, given its relation to increased parental depression symptoms, early weaning and increased risk of Shaken Baby Syndrome. To counteract the disadvantages of using a subjective outcome measure, we are also using a validated behaviour diary to obtain measures of sleep and cry duration. In a previous study of 446 infants, we found that diary data including total crying time and number of bouts of crying/24 hours were significantly increased in parents who reported problem crying in their infant vs. those who did not, whilst sleep duration and number of bouts of sleep/24 hours were significantly reduced in infant's whose parents reported a sleep problem vs. those who did not [5].

### Intervention

All parents allocated to the intervention group will be posted a study-designed booklet and DVD and will be offered an individual telephone consultation (at infant age 6 weeks) and group session (at infant age 12 weeks). Telephone consultations and parent groups will be facilitated by trained health professionals (nurses, psychologists) with a background in infant care. These facilitators will spend a minimum of two hours completing specific training in how to use the Baby Business telephone and group session manuals. This training will be facilitated by either the Chief Investigator (HH) or the project manager (FC), and all facilitators will observe a minimum of two telephone consultations and two groups before independently delivering program content.

### Booklet

The 27-page booklet provides parents with information about normal infant sleep patterns, sleep cycles and therefore, the potential for an infant to wake overnight several times. The benefit of an infant learning to fall asleep independently is discussed. Content highlights the disadvantages of an infant relying on parent dependent cues to fall asleep at the beginning of the night (e.g. by rocking). The dangers of parents sharing their bed with a newborn are also described. Steps to settling an infant are given with emphasis on the importance of putting the infant down for a sleep drowsy but awake, so that the infant can learn to fall asleep independently. Normal infant crying patterns are discussed (including the 'crying curve' showing the natural peak and subsequent decline in infant crying; used with permission from the website: http://www.purplecrying.info[57]) together with strategies for managing infant crying in both checklist and pictorial form. Signs and symptoms of uncommon medical causes of crying are outlined. Information on improving parental wellbeing is provided, as well as information on typical sleep and feeding patterns in Australian children after the first three months of life.

### DVD

The DVD covers very similar content to the booklet but also includes footage of parents discussing the methods they use to settle their infant, the tired signs their infant displays, as well as demonstrations of how they wrap and sooth their infant.

### Telephone consultation

The telephone consultation expands on the content of the booklet and DVD. During the telephone call, a facilitator helps the parent apply the information in a way that is suitable for their family. Parents are encouraged to discuss topics such as: whether they have noticed their infant's sleep cycles; how their infant behaves when over-tired and how to recognise and avoid over-tiredness; the advantages of teaching an infant to fall asleep without hands on help; the risks of co-sleeping; changes they would like to make to their settling routine as well as strategies they will use when their infant is having trouble settling; where their infant currently lies on the 'crying curve' and what they might expect to happen to crying duration in the coming weeks; colic; and, if appropriate, uncommon medical causes of crying.

### Parent Group

Parents are invited to attend a 1.5 hour group session at a local venue. The group aims to troubleshoot any problems parents are having with infant sleep and crying. The group facilitator follows a manual that covers daytime feeding and sleeping patterns at 3 to 4 months of age, day time napping and how to encourage longer daytime sleeps, night time feeding, use of dummies, wrapping, myths around infant care, and the importance of parental self-care.

### Usual Care

Families allocated to the 'usual care' (control) group receive no intervention from our research team. These families continue to receive the usual assistance and advice provided to all parents of newborns, via usual contact with MCH nurses and other health professionals.

### Measures

#### Baseline questionnaire

Parent A (the primary caregiver) will complete a baseline questionnaire including infant date of birth, birth weight, birth order, gestation, the type of milk being given, approximate number of feeds during the day and the night, caregiver's date of birth, country of birth, the main language spoken in the home, current marital status and highest level of education completed. Details are also gathered regarding partner date of birth, country of birth and their highest level of education completed. They will also be asked where their infant spends most of the night (parents bed, own cot/bed in other room, own cot/bed in parent's room or 'other') and whether their infant has been diagnosed with or suspected of any major illness.

Parent A will complete the 'doubt' subscale of the Maternal Cognitions about Infant Sleep Questionnaire [25] (see below). Other subscales are not relevant at such a young age. Feelings of parental doubt may already manifest at 2 to 4 weeks of age and such doubts are associated with increased likelihood of infant sleep problems [25]. Parent A will also be asked whether they think they are a relaxed or tense person (0 = relaxed to 10 = tense; Sayers, 2004); being 'tense' (scoring 7 or higher) predicts higher infant irritability at nine weeks of age.

#### Follow up questionnaires

In order to establish the efficacy of this intervention, we are measuring outcomes at infant age four and six months, since most infants 'sleep through the night' by four months of age [58] and no longer require night time feeding to meet their nutritional needs by six months of age. Measurement at these time points will therefore indicate established sleep patterns of these infants.

Both Parent A and Parent B (secondary caregiver) will complete follow up questionnaires at infant age four and six months. Only Parent A will complete an infant behaviour diary and the feeding questions at these time points.

### Primary & secondary outcomes

Our primary outcome is parent report of infant night time sleep problems. Secondary outcomes include parent report of day time sleep problems, infant crying problems and feeding problems. Parents will be asked 'Have any of the following baby behaviours been a problem for you over the last 2 weeks? Daytime sleep (yes/no), Night-time sleep (yes/no), Crying (yes/no)' [59]. If a parent answers 'yes' to any of these, they will rate the severity of the problem on a seven point Likert scale from 1 = 'hardly any problem' to 7 = 'a severe problem' [43].

Parent A will also complete an infant behaviour diary over a 72 hour period, during which they will record whether their infant is sleeping, feeding, awake and content or awake and crying/fussing in 10-minute epochs. This diary has been adapted from the Barr diary which measures such behaviours in 5 minute epochs and has sound psychometric properties [60].

### Parental sleep quality and quantity

Two items have been adapted from the validated Pittsburgh Sleep Quality Index (PSQI) [61] to measure parent perception of sleep quantity and quality: (1) 'Over the last two weeks, how would you rate your own sleep quantity?' with responses of 'Not nearly enough', 'Not quite enough', 'Enough' or 'More than enough' and (2) 'Over the last two weeks, how would you rate your own sleep quality?' with responses 'Not nearly good enough', 'Not quite good enough', 'Good enough' or 'More than good enough'.

Parents will also report how many times and for how long on average they attended their infant for night waking over the past week.

### Postnatal depression

The Edinburgh Postnatal Depression Scale (EPDS) [62] is a validated screen of postnatal depression (PND) [63,64] consisting of 10 items. Clinically significant levels of PND are indicated by scores ≥ 10 and ≥ 9 for mothers and fathers in community samples, respectively.

### Parental cognitions around infant sleep

The 20-item Maternal Cognitions about Infant Sleep Questionnaire (MCISQ) [25] has 5 subscales including: limit setting (ability to resist infant demands), anger (anger, regret and helplessness), doubt (uncertainty regarding ability as a parent), feeding (belief about the importance of feeding to settle and concerns about child going hungry) and safety (concerns about cot death). We have not included the 'feeding' subscale as it is not associated with infant sleep problems [25].

### Parental perception of infant temperament

Parents will rate their infant's temperament on a six point scale: 'Compared to other babies, I think my baby is:' Much easier than average, Easier than average, Average, More difficult than average, Much more difficult than average or cannot say. This single item from the Australian Temperament Project-a longitudinal study of 2000 children-has a moderate correlation with the Australian version of the Revised Infant Temperament Questionnaire [65].

### Sources of alternate help for infant sleep/crying

Parents will be asked if they have received help or advice (professional or otherwise) from outside the program for either their infant's sleep/crying or for their own stress, and if so, how many appointments they attended for each.

### Feeding

Parent A will indicate the type of food being offered to their infant at present (breast milk, formula or a combination of these) [66,67], and if applicable, whether breast feeding has stopped and why, whether their infant's formula has changed at any stage and whether the mother has changed her own diet while breastfeeding. The 6 month questionnaire also asks about timing of introduction and type of solids.

### Caregiver support and self-efficacy

At baseline and follow up, parent A will rate the level of support or help they receive from their partner and family and friends living elsewhere (I get enough help, I don't get enough help, I don't get any help, I don't need any help), and how often they feel they need support or help but can't get it from anyone (very often, often, sometimes, never, I don't need it) (LSAC) [68]. Parent A will rate their efficacy as a parent (from 1 = not very good to 5 = a very good parent) [68].

### Parental use of intervention materials

Intervention parents will be asked which components of the intervention they received or participated in and usefulness of each, on a study-designed scale ('not at all (useful), a little, quite a bit, a great deal'). Intervention parents will also be asked to rate the helpfulness of specific strategies provided in the intervention materials as either helpful or unhelpful.

### Procedures

Throughout the recruitment phase, MCH nurses approached all families of newborn infants at the first home visit and asked permission to pass on to the research team the contact details of families interested in hearing more about the research. The research team contacted interested families to explain the study further and posted a recruitment pack (information statement, consent form and baseline questionnaire) to parent A and an information pack (information statement and consent form) to parent B if requested. Families were randomised upon receipt of their signed consent form and baseline questionnaire.

Families allocated to the usual care group received a letter explaining that they will continue to see their MCH nurse as usual. Those allocated to the Baby Business intervention group were mailed the booklet and DVD.

A member of the study team completes the telephone consultation with intervention group parents one to two weeks after sending the booklet and DVD. Families are then invited to attend a parenting group session when their infant is around 12 weeks of age. Neither the telephone consultation nor the parent group is compulsory, and parents who do not take part in these components will not be removed from the study given that they will already have received most of the program content via the booklet and DVD. Data will be gathered on participation in each component.

In order to minimize study drop-out, we will call parents if they have not returned their follow up questionnaires two weeks after they were mailed to them. If we do not receive the completed questionnaire in the two weeks following this, we will call the parent again and give the parent the option of completing a shortened version of the questionnaire (covering only the main outcome measures of whether infant sleep, crying and feeding are a problem) over the phone.

### Hypotheses

We hypothesise that intervention parents compared to control group parents will, at infant age four and six months, report:

• fewer infant night time sleep problems (primary outcome)

• fewer infant day time sleep problems

• fewer crying problems

• decreased mean infant crying duration and increased mean infant sleep duration/24 hours

• improved parent sleep quality and quantity,

• improved mental health with lower mean EPDS scores and lower proportions of mothers scoring > 10 and fathers scoring > 9 on the EPDS

• less difficult infant temperament,

• fewer visits to healthcare professionals for their infant's sleep and crying and their own wellbeing

• higher rates of breastfeeding, fewer formula changes and fewer dietary changes in breastfeeding mothers

#### Analysis plan

Outcome data at 4 and 6 months will be presented using descriptive statistics. Means and standard deviations will be given for continuous outcomes, as well as medians and inter-quartile ranges where continuous data are skewed. Proportions for categorical data will also be given. The primary outcome comparison will be of the proportion of infants with sleep and cry problems at 4 months between the two trial arms. Logistic regression adjusting for potential confounders identified a priori, and measured at baseline (including child gender and family socioeconomic status), will be used to estimate the treatment effect as an odds ratio and 95% confidence interval. Random effects regression models [69,70] will be used for further longitudinal analysis examining trends in treatment response, that is, persistence of sleep and cry problems from baseline to 4 and 6 months post intervention. Similar analysis will be carried out for each of the categorical outcomes. We will also compare mean scores for continuous outcomes at the primary endpoint of 4 months (e.g. sleep duration/24 hours) between the two trial arms using t tests, as well as linear regression adjusting for potential confounders. The study sample size is sufficient to enable the use of such techniques when the outcome data are skewed [71], and empirical bootstrap estimates will be examined to confirm the validity of the inferences made. Trends in treatment response will again be examined from baseline to 4 and 6 months post intervention using random effects regression models. All analyses will be conducted on the basis of intention to treat. The frequency and patterns of missing data will be examined and sensitivity analyses will be performed comparing the results of analyses restricted to families with complete data and analyses where missing data are imputed using a conservative approach [72].

#### Economic evaluation

A cost-consequences analysis will be conducted from a societal perspective. Costs and outcomes will be taken into account and valued in the analysis regardless of who bears the costs, who benefits or who provides the resources. The incremental costs of the intervention (the difference of costs accrued in the intervention group and costs accrued in the control group) will be compared to a range of the incremental primary and secondary outcomes. Both costs of delivering the intervention and costs of families' use of health and other services outside of the study will be considered in economics costing.

The economic evaluation will draw a comprehensive picture of the costs and consequences of the intervention and assist policy makers to make appropriate decisions on resource allocation to such interventions and determine the cost-effectiveness of nationwide roll-out.

### Ethical approval

Ethical approval has been obtained from the Royal Children's Hospital Human Research Ethics Committee (HREC 28130) and the Department of Education and Early Childhood Development, Early Childhood Research Committee.

## Competing interests

The authors declare that they have no competing interests.

## Authors' contributions

FC and HH drafted the manuscript, with JB, HL, FM and WC contributing to several revisions. HH and JB are responsible for the study design. All authors have given approval for the final version to be published.

## Pre-publication history

The pre-publication history for this paper can be accessed here:

http://www.biomedcentral.com/1471-2431/12/13/prepub
